# Memory reconsolidation in aversive and appetitive settings

**DOI:** 10.3389/fnbeh.2013.00118

**Published:** 2013-09-09

**Authors:** Amy C. Reichelt, Jonathan L. C. Lee

**Affiliations:** School of Psychology, University of BirminghamBirmingham, UK

**Keywords:** memory reconsolidation, fear memory, appetitive conditioning, drug addiction, post-traumatic stress disorder, NMDA antagonists, protein synthesis inhibitors

## Abstract

Memory reconsolidation has been observed across species and in a number of behavioral paradigms. The majority of memory reconsolidation studies have been carried out in Pavlovian fear conditioning and other aversive memory settings, with potential implications for the treatment of post-traumatic stress disorder. However, there is a growing literature on memory reconsolidation in appetitive reward-related memory paradigms, including translational models of drug addiction. While there appears to be substantial similarity in the basic phenomenon and underlying mechanisms of memory reconsolidation across unconditioned stimulus valence, there are also notable discrepancies. These arise both when comparing aversive to appetitive paradigms and also across different paradigms within the same valence of memory. We review the demonstration of memory reconsolidation across different aversive and appetitive memory paradigms, the commonalities and differences in underlying mechanisms and the conditions under which each memory undergoes reconsolidation. We focus particularly on whether principles derived from the aversive literature are applicable to appetitive settings, and also whether the expanding literature in appetitive paradigms is informative for fear memory reconsolidation.

## Introduction

Memories are dynamic, as opposed to static, in nature. Instead of remaining in a stable, permanent state, memories can be strengthened, weakened or modified. Memory reconsolidation is proposed to be an important mechanism of memory modification, ensuring that the memory maintains relevance to current and future behaviors (Lee, 2009). Memory reconsolidation is the process by which memories that have been destabilized by retrieval are destabilized (Nader et al., 2000b; Nader, 2003; Dudai, 2006). Thus, memories may be retrieved (or reactivated) in an environment presenting additional information; this can cause the memory to enter into an unstable, labile state that is susceptible to change. Classically, this has been exploited by applying amnestic agents [such as electric shock, protein synthesis inhibitors, metabolic process blockers or amnesic agents (Misanin et al., 1968; Nader et al., 2000a; Lee et al., 2005)] either prior to or shortly after memory reactivation that disrupt memory destabilization in order to produce an experimental amnesia that is demonstrative of the existence of reconsolidation. However, in the absence of amnestic treatment the new information is thought to be adaptively incorporated within the pre-existing memory trace (Dudai and Eisenberg, 2004; Dudai, 2006; Tronson and Taylor, 2007; Lee, 2009).

Reconsolidation of memories has been observed in many species including invertebrates such as nematodes (Rose and Rankin, 2006), honeybees (Stollhoff et al., 2005, 2008), sea slugs (Child et al., 2003; Cai et al., 2012; Lee et al., 2012), and crabs (Pedreira et al., 2002; Carbo Tano et al., 2009) and vertebrates including mice (Kida et al., 2009), rats (Nader et al., 2000a), humans (Hupbach et al., 2007, 2009), sheep (Perrin et al., 2007), rabbits (Coureaud et al., 2009) and chicks (Hale and Crowe, 2003). Reconsolidation has most frequently been studied in laboratory rodents using aversive Pavlovian conditioning paradigms utilizing both cued (Nader et al., 2000a) and contextual fear (Debiec et al., 2002; Lee, 2008), as well as conditioned taste aversion (CTA) (Garcia-Delatorre et al., 2010) and inhibitory avoidance (Boccia et al., 2005, 2011; Inda et al., 2011). These paradigms are well suited to the study of learning and memory, given their rapid acquisition, and have been especially useful in addressing the question of the mechanistic similarity between initial consolidation and subsequent reconsolidation (Alberini, 2005). The continued focus on Pavlovian fear conditioning is driven, at least in part, by the translational potential reconsolidation-based treatment strategies for human anxiety disorders including phobias and post-traumatic stress disorder (PTSD) (Debiec and Altemus, 2006; Brunet et al., 2008, 2011b).

The demonstration of memory reconsolidation has not been limited to aversive memory settings, and reconsolidation impairments have also been observed in a number of non-aversive experimental paradigms including spatial memory (Morris et al., 2006), object recognition (Akirav and Maroun, 2006; Rossato et al., 2007), odor discrimination (Portero-Tresserra et al., 2013) and appetitive Pavlovian memories with both natural and addictive drug rewards (Lee and Everitt, 2008a; Milton et al., 2008b). The latter of these is of particular interest, given the therapeutic potential of disrupting the reconsolidation of Pavlovian cue-drug memories as a pro-abstinence/anti-relapse treatment for drug addiction (Tronson and Taylor, 2007; Milton et al., 2012). The intensive research on reconsolidation in both aversive and appetitive memory settings, especially with their relevant translational foci, raises the important question of to what extent do the findings from each field generalize across experimental paradigms. This review will therefore, present mechanistic principles of appetitive and aversive memory reconsolidation, highlighting the similarities and differences between these paradigms.

## Behavioural phenomenology of reconsolidation

Given that the existence of memory reconsolidation can only be inferred from its disruption, the application of an amnestic agent is often critical to reconsolidation studies (the alternatives being to assess the impact of interfering material (Hupbach et al., 2009; Caffaro et al., 2012) and stress (Wang et al., 2008; Schwabe and Wolf, 2010). From such studies, we can derive an understanding of the pharmacological and cellular mechanisms of memory reconsolidation in each setting. As the mechanisms of reconsolidation have been reviewed elsewhere (Tronson and Taylor, 2007; Nader and Hardt, 2009), we will restrict ourselves to the consideration of evidence from a selection of pharmacological and cellular interventions in order to highlight the commonalities and differences between the reconsolidation of aversive and appetitive memories at both the mechanistic and phenomenological levels. Memory reconsolidation is evoked through the reactivation or recall of a specific memory, this reactivation process causes the memory to become destabilized into a so called “labile” state. The destabilization process to return this memory into a stable form is known as reconsolidation. Amnestic agents can be applied both prior to or immediately following memory reactivation, leading to a disruption of the reconsolidation process and evoking amnesia for the original memory. Moreover, the amnestic treatments, regardless of their nature, allow an understanding of the behavioral phenomenology of memory reconsolidation. By this, we mean the underlying principles and functions of the reconsolidation process. Within this context, we will focus on two questions in particular:

1. Does reconsolidation take place for all types of memories?
  While this question cannot be answered definitively, we will focus on aversive and appetitive associative memories that are important from a translational perspective. The demonstration of the existence of memory reconsolidation across paradigms is an important first step in assessing the generality of findings observed in any given setting. We will also highlight salient mechanistic commonalities and differences between paradigms.
2. What determines whether or not a memory undergoes reconsolidation?
  From a translational perspective, the efficacy with which a maladaptive memory can be reactivated such that its reconsolidation can be disrupted is a critical issue. Much of the relevant research has been conducted in aversive settings, but there are salient comparisons to be made with appetitive studies.

## Does reconsolidation take place for all types of memories?

Even restricting our review to aversive and appetitive Pavlovian conditioning leaves us considering a number of behavioral paradigms. In the aversive domain, rodent studies have employed fear conditioning to both discrete and contextual cues, inhibitory avoidance and CTA. By comparison, there is also a number of different appetitive conditioning procedures whereby the unconditioned stimulus (US) is a natural reward such as food, in which reconsolidation has been observed as reviewed in the following section. We focus principally on research that has targeted gene expression, protein synthesis, *N*-Methyl-D-aspartate receptors (NMDAR) and β-adrenergic receptors and these are outlined in Table 1. The use of drugs, both systemic and site specific, that interfere with these cellular and pharmacological mechanisms has been widespread in aversive and appetitive memory reconsolidation studies, allowing a degree of mechanistic comparison.

**Table 1:** **Summary of aversive and appetitive paradigms including details of target structures and mechanisms**.

**Setting**	**Behavioural paradigm**	**Manipulation**	**Target region**	**References**
Aversive	*Disruption—Amnesia*	Protein synthesis inhibition—Anisomycin	Lateral amygdala	Nader et al., 2000a; Duvarci and Nader, 2004
	Cued fear conditioning			
		NMDA antagonism—MK-801	Systemic	Ben Mamou et al., 2006; Lee et al., 2006b
			BLA	Ben Mamou et al., 2006
		B-adrenergic antagonism—propranolol	Lateral amygdala	Debiec and Ledoux, 2004, 2006
			Systemic	Kindt et al., 2009; Soeter and Kindt, 2012; Kindt and Soeter, 2013
		Protein kinase A inhibitor	Lateral amygdala	Tronson et al., 2006
		Cannabinoid CB1 agonist—WIN55212-2	BLA	Lin et al., 2006
	Contextual fear conditioning	Protein synthesis inhibition—Anisomycin	Hippocampus	Debiec et al., 2002
		Transcription factor inhibition—Zif268	Hippocampus	Lee et al., 2004
			Amygdala	Lee et al., 2004; Malkani et al., 2004; Maddox et al., 2011
		NMDA antagonism—AP5	Hippocampus	Lee and Hynds, 2012
		NMDA antagonism—MK-801	Systemic	Charlier and Tirelli, 2011
		β-adrenergic antagonism—Propranolol	Systemic	Abrari et al., 2008
		GABA_A_ agonist - midazolam	Systemic	Bustos et al., 2006; Zhang and Cranney, 2008
	Inhibitory avoidance	Protein synthesis inhibition—Anisomycin	Amygdala	Milekic et al., 2007
		β-adrenergic antagonism—Propranolol	Systemic	Przybyslawski et al., 1999
		Acetylcholine antagonist—α 7 nicotinic antagonist	Hippocampus	Boccia et al., 2010
		Transcription inhibitor—NF-κB	Hippocampus	Boccia et al., 2007; Jobim et al., 2012
		mTOR inhibition	Hippocampus and BLA	Jobim et al., 2012
			BLA	Pedroso et al., 2013
	Conditioned taste aversion	Protein synthesis inhibition—Cyclohexamide	Systemic	Flint and Marino, 2007
		Anisomycin	Insular cortex	Eisenberg et al., 2003; Bahar et al., 2004
		Protein kinase inhibition	BLA	Koh and Bernstein, 2003
		Cannabinoid CB1 agonist—WIN55212-2	Insular cortex	Kobilo et al., 2007
	*Enhancement*	NMDA agonism—d-cycloserine	Systemic	Lee et al., 2006b
	Cued fear conditioning	Cannabinoid CB1 agonist—WIN55212-2	Hippocampus	de Oliveira Alvares et al., 2008
	Contextual fear conditioning	NMDA agonism—d-cycloserine	Systemic	Yamada et al., 2009
Appetitive	*Disruption—Amnesia*	Protein synthesis inhibition—Anisomycin	BLA	Lee et al., 2005
	Conditioned reinforcement			
		Transcription factor inhibition—Zif268	BLA	Lee et al., 2005
		NMDA antagonist—AP5	BLA	Milton et al., 2008a
		MK-801	Systemic	Lee and Everitt, 2008a
		β-adrenergic antagonism—Propranolol	Systemic	Milton et al., 2008b
	Sign-tracking and Pavlovian—Instrumental transfer	NMDA antagonist—MK-801	Systemic	Lee and Everitt, 2008a,c; Milton et al., 2008a, 2012
		B-adrenergic antagonism—Propranolol (cocaine sign tracking only)	Systemic	Milton and Everitt, 2010
	Goal tracking	NMDA antagonist—MK-801	Systemic	Reichelt and Lee, 2012
	Conditioned place preference	Protein synthesis inhibition—Anisomycin	BLA	Milekic et al., 2006
		NMDA antagonist—AP5	Hippocampus	Sakurai et al., 2007
		MK-801	Systemic	Sadler et al., 2007; Brown et al., 2008; Alaghband and Marshall, 2012
		β-adrenergic antagonism—Propranolol	Systemic	Bernardi et al., 2006; Robinson and Franklin, 2007a,b; Fricks-Gleason and Marshall, 2008; Achterberg et al., 2012
		GABA_A_ agonist—Midazolam	Systemic	Robinson and Franklin, 2010
		Muscarinic acetylcholine agonist—Scopolamine	Systemic	Kelley et al., 2007
		Transcription inhibitor—NF-κB	Intraventricular	Yang et al., 2011
		-cdK5	BLA	Li et al., 2010
		-ERK	Frontal cortex	Li et al., 2008
	Odor-reward discrimination	NMDA antagonist—AP5	Intraventricular	Torras-Garcia et al., 2005
	*Enhancement*	NMDA agonist—d-cycloserine	BLA	Lee et al., 2009
	Conditioned reinforcement			
	Odor-reward discrimination	NMDA agonist—d-cycloserine	BLA	Portero-Tresserra et al., 2013
	Conditioned place preference	NMDA agonist—d-cycloserine	Systemic	Kelley et al., 2007

### Aversive memory reconsolidation

#### Discrete cued fear conditioning

The earliest demonstration of what is now considered to be memory reconsolidation used fear conditioning of an aversive footshock US to a discrete white noise conditioned stimulus (CS), with electroconvulsive shock (ECS) as the treatment (Misanin et al., 1968). This experiment demonstrated what the authors described as “cue dependent amnesia,” when ECS immediately followed an aversive CS-US pairing, the memory for the pairing was disrupted. However, when the ECS was delayed until 24 h post-noise-shock pairing, no disruption was observed. It was suggested that ECS given shortly after training disrupted the newly-formed active memory that was then unable to consolidate properly, resulting in impaired memory for the noise-shock pairing. However, when the ECS was applied a day later, the memory was in a consolidated, inactive state. Crucially, it was observed that when a noise-shock pairing had been learned, presentation of the CS again prior to ECS application resulted in disrupted memory. This study importantly demonstrated that presentation of the CS reactivated and destabilized the memory of a noise-shock pairing, leaving the memory susceptible to disruption.

The recent increase in research into memory reconsolidation can be attributed to the study by Nader et al. (2000a) and its demonstration that fear memory reconsolidation can be disrupted by localized intracerebral infusions of amnestic agents. Specifically, they demonstrated that conditioned fear can be disrupted by blocking reconsolidation using infusion of the protein synthesis inhibitor anisomycin into the lateral amygdala (LA). In this study, rats were conditioned to fear a tone CS. Following conditioning, rats were presented with an unreinforced presentation of the CS, which served to reactivate and destabilize the fear memory trace. This reactivation was followed immediately by infusion of anisomycin or saline into the LA. At test, the rats that received anisomycin after reactivation showed greatly reduced freezing to the CS, indicating that protein synthesis is necessary for the successful destabilization of memories during reconsolidation. The control rats that received either a saline infusion after reactivation of the CS or anisomycin without reactivation continued to freeze indicating normal conditioned fear. This study demonstrated that the reconsolidation of fear memories is critically dependent upon *de novo* protein-synthesis and this appears to be regulated, at least in part, but mTOR activity. When a mammalian target of rapamycin kinase (mTOR) inhibitor was infused into the amygdala after the recall of a previously stored cued fear memory, subsequent retention was disrupted (Parsons et al., 2006), indicating that memory reconsolidation is dependent on both protein synthesis and mTOR activity within the BLA. In addition, further studies indicated that the disruption of reconsolidation was specific to the fear memory reactivated, leaving other memories intact (Doyere et al., 2007), and that the disruption of the fear memories was enduring in nature, not returning following the alteration of contextual cues, after the passage of time, or additional stress (Duvarci and Nader, 2004).

Among other mechanisms, fear memory reconsolidation has also been shown to depend upon NMDAR involvement (Ben Mamou et al., 2006; Lee et al., 2006b). Systemic injection of the NMDAR antagonist [5R,10S]-[+]-5-methyl-10,11-dihydro-5H-dibenzo[a,d]cyclohepten-5,10-imine (MK-801) impaired reconsolidation of a conditioned fear memory resulting in reduced freezing and in contrast injection of the partial agonist D-cyloserine (DCS) increased freezing to the CS (Lee et al., 2006b). This study therefore, indicated a role of NMDA receptors in the disruption and enhanced expression of fear following reactivation of conditioned fear. In addition, the activation of NMDA receptors in the region of the basolateral amygdala (BLA) was demonstrated to be crucial in the destabilization of fear memories (Ben Mamou et al., 2006). Infusion of the NMDA NR2B subunit antagonist ifenprodil into the BLA protected the memory from anisomycin's amnesic effects, indicating that if destabilization of a memory is impaired, reconsolidation does not occur. It remains unclear why systemic MK-801 impairs reconsolidation, while intra-amygdala (2*R*)-amino-5-phosphonopentanoate (AP5) impairs destabilization. Potential explanations may involve either effects of MK-801 in extra-amygdala neural loci, or the differing pharmacological nature of AP5 (competitive antagonist) compared to MK-801 (non-competitive antagonist) (Milton et al., 2013).

Cued-fear memory reconsolidation has been associated not only with NMDA receptors, but also β-adrenergic receptors (β-AR). Infusion of the β-AR antagonist propranolol into the lateral nucleus of the amygdala (LA) was shown to disrupt memory reconsolidation following retrieval (Debiec and Ledoux, 2004, 2006). Similarly, stimulation of noradrenergic receptors in the LA with a β-AR agonist was shown to enhance fear (Debiec et al., 2011). Further mechanisms and demonstration of bi-directional modulation of reconsolidation include protein kinase A (PKA) whereby the activation of amygdalar PKA was sufficient to enhance memory following retrieval and PKA inhibition impaired reconsolidation (Tronson et al., 2006).

#### Contextual fear conditioning

While contextual fear memories share a dependence upon the amygdala with discrete fear memories (Phillips and Ledoux, 1992; Bergstrom et al., 2012; Flavell and Lee, 2012), they also demonstrate that hippocampal memory traces undergo reconsolidation. Infusion of anisomycin into the dorsal hippocampus following memory reactivation was shown to reduce contextual fear at test (Debiec et al., 2002), demonstrating the common dependence of hippocampal memory reconsolidation and consolidation (Vianna et al., 2001) upon *de novo* protein synthesis. Furthermore, this *de novo* protein synthesis is controlled by mTOR, as evidenced by the common effect of rapamycin on both the consolidation and reconsolidation of a contextual fear memory (Gafford et al., 2011). However, independent cellular processes have been observed within the hippocampus in the consolidation and reconsolidation of contextual fear memories (Lee et al., 2004). This finding importantly demonstrated a double dissociation between the transcription factors brain-derived neurotrophic factor (BDNF) and Zif268. It has been noted whereby new learning (consolidation) of contextual fear memories was shown to depend upon BDNF, whereas reconsolidation was shown to require Zif268 within the hippocampus (Lee et al., 2004). This dissociation may be unique to the hippocampus as Zif268 has been implicated in both consolidation and reconsolidation in the amygdala (Malkani et al., 2004; Lee et al., 2005; Maddox et al., 2011).

Upstream of *de novo* protein synthesis, diverging pathways of hippocampal contextual fear memory consolidation and reconsolidation have been observed. While the double dissociation between BDNF and Zif268 expression extended to the selective requirement for the MEK and IKKα protein kinases in consolidation and reconsolidation, respectively, there was a common involvement of NMDA receptors (Lee and Hynds, 2012). Infusion of AP5 directly into the dorsal hippocampus impaired both acquisition/consolidation and reconsolidation. Unlike in the amygdala, there is no discrepancy between the effects of local AP5 infusions and systemic MK-801 injection, as the latter also impaired contextual fear memory reconsolidation in mice (Charlier and Tirelli, 2011). Similarly, the systemic injection of propranolol moderately interferes with the reconsolidation of contextual Pavlovian fear memories (Abrari et al., 2008), although it has yet to be determined whether local infusions of propranolol directly into the hippocampus also disrupt contextual fear memory reconsolidation. Moreover, systemic application of the GABA_A_ receptor agonist midazolam, an anxiolytic drug, was shown to disrupt contextual fear memory following reactivation (Bustos et al., 2006; Zhang and Cranney, 2008). It has, however, yet to be demonstrated whether or not midazolam or other GABAergic agonists have similarly-disruptive effects on cued fear memory reconsolidation. Therefore, the mechanisms of hippocampal contextual fear memory reconsolidation are largely similar at the cell surface level to those of amygdala cued fear memory reconsolidation. However, there appear to be salient discrepancies at the level of intracellular signaling cascades and gene expression.

#### Inhibitory avoidance

Inhibitory avoidance (IA) protocols measure behavioral preference for a safe environment as opposed to an environment paired with footshock. Systemic β-AR antagonism has been shown to impair memory for the shock paired context in IA tasks (Przybyslawski et al., 1999), indicating a therapeutic role in the treatment of fear memory discorders. It has been noted that intra-hippocampal infusion of anisomycin does not impair the reconsolidation of IA memories (Taubenfeld et al., 2001; Power et al., 2006), however, Milekic et al. (2007) demonstrated that intra-amygdala anisomycin did impair IA memory reconsolidation. Despite the widespread use of anisomycin as the prototypical treatment for the demonstration of reconsolidation impairments, the failure of intra-hippocampal anisomycin to disrupt memory reconsolidation in IA does not seemingly rule out a role for hippocampal plasticity in IA memory reconsolidation. There are numerous demonstrations that infusions of substances directly into the dorsal hippocampus impaired IA memory reconsolidation. For example, intra-hippocampal application of cholinergic antagonists in mice resulted in a reactivation dependent impairment of IA memories (Boccia et al., 2010). Moreover, transcription in the hippocampus appears to be important for the reconsolidation of IA memories. Both the transcriptional inhibitor rapamycin and inhibition of the transcription factor NF-κB in the hippocampus impaired reconsolidation of IA memories (Boccia et al., 2007; Jobim et al., 2012). This protein synthesis appears to be regulated, at least in part, but mTOR activity. Recently, (Pedroso et al., 2013) demonstrated that inhibition of mTOR activity in the BLA blocked retrieval-induced strengthening of an inhibitory avoidance memory, indicating that memory reconsolidation is dependent on protein synthesis and mTOR activity within the BLA.

The contrast between the necessity for gene transcription and the apparent lack of requirement for mRNA translation in IA memory reconsolidation is not easily reconciled. It has previously been argued that a higher dose of anisomycin is necessary to impair hippocampal memory reconsolidation than that which was ineffective for IA (Debiec et al., 2002). Alternatively, mTOR and NF-κB might play functional roles that do not require downstream mRNA translation. Regardless, the apparent functional activation of mTOR and NF-κB likely necessitates upstream processes, although whether these include NMDAR and/or β-AR has yet to be investigated. What has been demonstrated, however, is that propranolol has limited reconsolidation disrupting properties in an IA setting when administered systemically, even though it was capable of disrupting Pavlovian fear memories (Muravieva and Alberini, 2010).

As noted previously, the reconsolidation if IA memories does require de novo protein synthesis in the BLA (Milekic et al., 2007). Moreover, the mTOR inhibitor rapamycin impaired IA memory reconsolidation not only when infused into the dorsal hippocampus, but also upon intra-BLA infusion (Jobim et al., 2012). Again, there is little more known about the mechanisms of IA memory reconsolidation in the BLA, with no reported effects or otherwise of cell surface receptor antagonists infused into the BLA. Therefore, it remains unclear to what degree the mechanisms of IA memory reconsolidation in the amygdala and hippocampus are consistent with those observed for cued and contextual fear memory reconsolidation.

#### Conditioned taste aversion

Studies of CTA also appear to reveal that the infusion of protein synthesis inhibitors is not sufficient to determine the functional involvement of a brain region in the plasticity of memory reconsolidation. CTA involves the pairing of a taste CS with an US capable of inducing general malaise, such as pairing a flavored liquid with lithium chloride (LiCl). Subsequently the aversive association with sickness leads to avoidance of the CS. CTA memories can be reactivated by re-exposing the animals to the taste CS, and systemic administration of the protein synthesis inhibitor cyclohexamide was shown to increase consumption of the sickness-paired CS, indicative of reactivation dependent amnesia (Flint and Marino, 2007). In a choice setting whereby one liquid (saccharin) was paired with sickness induced by LiCl over 2 days, aversion indices demonstrated that rats which received anisomycin into the insular cortex following reactivation drank more saccharin than control rats (Eisenberg et al., 2003).

While the insular cortex has been reliably implicated in CTA memory reconsolidation, the involvement of the amygdala is less clear. Bahar et al. (2004) demonstrated that infusion of anisomycin into the BLA or central nucleus (CeN) had no effect on reconsolidation of CTA, while under the same conditions, intra-insular infusions did result in amnesia. However, inhibition of protein kinase A in the BLA did disrupt CTA memories in a retrieval dependent manner (Koh and Bernstein, 2003). Therefore, again it remains to be clarified what role cellular mechanisms play in selective loci (in this case, the BLA) if not ultimately leading to the synthesis of functionally-necessary new proteins.

At the cell surface level, the role of NMDARs and β-ARs in CTA memory reconsolidation have not yet been studied. However, it has been demonstrated that the cannabinoid CB1 receptor agonist WIN55212-2, when infused into the insular cortex, disrupted memory reconsolidation for CTA (Kobilo et al., 2007). This observation is similar to the findings of (Lin et al., 2006), who demonstrated that infusion of WIN55212-2 into the BLA impaired memory reconsolidation in a discrete cue fear-potentiated startle study. When it comes to contextual fear conditioning, however, the literature is more mixed on the functional role of CB1 receptors. While CB1 receptor antagonism in the dorsal hippocampus potentiated memory reconsolidation in rats (de Oliveira Alvares et al., 2008), studies by Suzuki and colleagues in mice have shown that a different CB1 receptor antagonist did not enhance reconsolidation (Suzuki et al., 2004), but rather impaired the destabilization of the contextual fear memory (Suzuki et al., 2004).

#### Aversive memory—summary

Memory reconsolidation has been demonstrated across a wide range of aversive Pavlovian conditioning procedures. However, it remains surprisingly difficult to draw firm conclusions in relation to common fundamental mechanisms of memory reconsolidation. At the cell surface, cued and contextual fear memories appear to require activity at NMDARs and β-ARs, although these have not been conclusively located to the hippocampus for contextual fear. Moreover, there is a lack of literature on the effect of NMDAR and β-AR antagonists on the reconsolidation of IA and CTA memories. Even for the canonical role of *de novo* protein synthesis in memory reconsolidation, there remains some confusion. While translational inhibitors impair the reconsolidation of conditioned fear memories in the amygdala and hippocampus, they are without effect in the same loci for CTA and IA, respectively. Nevertheless, evidence from other inhibitors of cellular function do implicate the amygdala in CTA memory reconsolidation and the dorsal hippocampus in IA memory reconsolidation.

### Appetitive memory reconsolidation

Fewer studies have been conducted investigating reconsolidation in appetitive memory paradigms, despite the importance of research into the modulation of established memories such as those formed during the course of drug addiction. The relapsing nature of drug addiction has been attributed to the presence of drug-associated cues in the environment that can trigger drug-seeking behaviors (Crombag and Shaham, 2002). The first demonstrations of non-aversive memory reconsolidation were demonstrated in separate settings; initially (Przybyslawski and Sara, 1997) demonstrated that an established spatial memory was reactivated by a single errorless trial and this was dependent upon NMDA receptors to maintain stability. Further studies (Roullet and Sara, 1998; Przybyslawski et al., 1999), indicated the importance of β ARs following the reactivation of established memories, in both spatial (food rewarded radial arm maze) and fear-driven tasks. These studies were the first to suggest the role of β AR antagonists in the treatment of fear-related disorders such as PTSD. Further early studies focused on appetitive paradigms, conditioned reinforcement in cocaine seeking behavior (Lee et al., 2005, 2006a), odor discrimination in a foraging for natural reward task (Torras-Garcia et al., 2005) and in CPP for cocaine (Miller and Marshall, 2005; Bernardi et al., 2006; Valjent et al., 2006). However, appetitive Pavlovian conditioning is complex and may involve multiple functional associations that can be isolated in specific experimental paradigms and it is only more recently that reconsolidation has been investigated in those settings. Memory reconsolidation processes have been shown to be involved in the maintenance of appetitive associations between stimuli and rewards such as cocaine (Lee et al., 2006a; Milton et al., 2008a), sucrose (Diergaarde et al., 2006; Lee and Everitt, 2008a) and alcohol (Wouda et al., 2010; Milton et al., 2012). These studies have been carried out in a number of paradigms including Pavlovian conditioned reinforcement (Lee and Everitt, 2008a; Milton et al., 2008b), sign-tracking (Lee and Everitt, 2008a; Milton et al., 2012), goal-tracking (Reichelt and Lee, 2012), but see (Blaiss and Janak, 2007), Pavlovian-instrumental transfer (PIT) (Lee and Everitt, 2008a; Milton et al., 2012), odor discrimination tasks (Portero-Tresserra et al., 2013) and conditioned place preference (CPP) (Miller and Marshall, 2005; Valjent et al., 2006; Robinson and Franklin, 2007a,b; Robinson et al., 2011a,b).

#### Conditioned reinforcement

Pairing of a neutral stimulus (i.e., a tone) with a valued reward (i.e., food) results in the formation of an association between the stimulus and outcome. The originally neutral stimulus becomes a CS and as a direct predictor of reward acquires conditioned reinforcing properties itself, being able to reinforce the acquisition of a new instrumental response (Kelleher and Gollub, 1962). It was within such a conditioned reinforcement setting that appetitive memory reconsolidation was first observed. In a study of cocaine seeking, infusion of anisomycin into the BLA immediately after memory reactivation impaired the subsequent acquisition of a new lever press response (Lee et al., 2005). Therefore, reconsolidation was shown to occur in the amygdala not only for aversive memories, but also for appetitive addictive drug-related memories. Moreover, there was a commonality of requirement for the synthesis of new proteins, including that of *Zif268* (Lee et al., 2005). As yet, however, there is no evidence whether or not the expression of *Zif268* is selectively necessary for reconsolidation, nor the functional involvement of BDNF in either consolidation or reconsolidation.

There is clear evidence that antagonism of NMDAR or β-adrenergic receptors impairs reconsolidation of the memory representations underlying conditioned reinforcement for both sucrose and cocaine primary rewards (Lee and Everitt, 2008a; Milton et al., 2008a,b). In sucrose seeking paradigms, systemic administration of MK-801 both prior to or following memory reactivation resulted in a reactivation-dependent impairment in the subsequent acquisition of a new response (Lee and Everitt, 2008a). While there has been no directly equivalent study in the cocaine-seeking setting, it has been demonstrated that infusions of the NMDA receptor antagonist AP5 directly into the BLA impair memory reconsolidation for cocaine conditioned reinforcement (Milton et al., 2008a). In contrast, it has yet to be demonstrated conclusively that the BLA is a primary central locus of effect of systemically-administered propranolol.

#### Sign-tracking and pavlovian-instrumental transfer

Sign-tracking, or autoshaping, procedures have been utilized to study the acquired incentive properties of a CS that has been associated with reward. When a spatially localized stimulus (such as the insertion of lever into a conditioning chamber or illumination of a stimulus-light) is predictive of a reward, animals will learn to respond not only to where the reward is delivered, but to the CS itself by approaching and contacting the location (Brown and Jenkins, 1968). Reconsolidation studies utilizing discrete CS presentations predictive of an appetitive outcome have indicated that the appetitive Pavlovian memory representations underlying autoshaping behavior can be disrupted through application of MK-801, but not propranolol, to induce reactivation dependent amnesia in rats trained to respond for sucrose (Lee and Everitt, 2008a). Additionally, conditioned approach for ethanol was demonstrated to be disrupted in a reactivation dependent manner by MK-801, but not propranolol (Milton et al., 2012).

Similarly, PIT procedures are used to assess the impact of Pavlovian conditioned stimuli upon instrumental performance (Hall et al., 2001). In this setting, rats are trained to respond to discrete CSs predictive of outcomes, and concurrently to produce instrumental responses reinforced with the same outcomes. At test, previously conditioned appetitive Pavlovian stimuli enhance the rate of instrumental responding, indicative of their influence on the motivational control of behaviors (Lovibond, 1983). Using a PIT method, the reconsolidation of the memories underlying these behavioral responses were impaired by the systemic administration of MK-801 at memory reactivation, but not the β-adrenergic receptor antagonist propranolol (Lee and Everitt, 2008a). Similarly, MK-801 application, but not propranolol, given in conjunction with reactivation was capable of disrupting PIT for ethanol associated cues (Milton et al., 2012).

While memory reconsolidation has been demonstrated to occur for the memories underlying autoshaping and PIT, as yet systemic injection of MK-801 is the only treatment shown to disrupt such reconsolidation. Therefore, the mechanisms of appetitive memory reconsolidation in these settings remain to be delineated including, importantly, the critical neural loci. While it has also not yet been determined whether memory reconsolidation requires *de novo* protein synthesis in these settings, the amnestic effect of MK-801 appears to be sufficient evidence for the existence of memory reconsolidation.

Considering the literature on conditioned reinforcement along with that on autoshaping and PIT, reconsolidation disruption has been observed across all appetitive paradigms with the NMDA receptor antagonist MK-801 (Lee and Everitt, 2008a; Milton et al., 2008a, 2012). However, the effect of propranolol disrupting sucrose and cocaine memory reconsolidation has only been observed on conditioned reinforcement (Milton et al., 2008b). As noted by Milton and Everitt (2010), conditioned reinforcement, autoshaping and PIT all contribute to relapse to reward-seeking behavior. Therefore, it is perhaps unsurprising that in preclinical models of relapse behavior; MK-801 successfully disrupts reconsolidation to reduce cocaine (Milton et al., 2008a) and sucrose (Lee and Everitt, 2008c) seeking. In contrast, propranolol was without equivalent effect in the cocaine-seeking setting (Milton et al., 2008b).

#### Goal-tracking

Goal-tracking behavior is acquired following pairings of a CS with a reinforcer, which leads to increased numbers of responses to the site of reinforcement whilst the CS is present (Costa and Boakes, 2009), and only recently have we demonstrated that the underlying memory representations undergo reconsolidation (Reichelt and Lee, 2012). Goal-tracking behaviors indicate that animals have learned to associate a discrete CS with the US, despite the absence of responding directed at the CS i.e., sign-tracking (Costa and Boakes, 2009). In an initial study of memory reconsolidation in a goal-tracking setting, Blaiss and Janak (2007) failed to find evidence that the underlying goal-tracking memory undergoes protein synthesis-dependent reconsolidation. However, we have demonstrated that that, under discrete conditions, pre-reactivation systemic MK-801 application impairs appetitive goal-tracking behavior (Reichelt and Lee, 2012). The discrepancies between the two studies may be indicative either of the insensitivity of goal-tracking memories to post-reactivation protein synthesis inhibition, or to only specific parameters of training and reactivation being sufficient to reactivate and destabilize goal-tracking memories. While it again remains to be seen whether protein synthesis inhibition can impair the reconsolidation of goal-tracking memories, the disruptive effect of MK-801 is consistent with the observations of other appetitive reconsolidation studies (Kelley et al., 2007; Sadler et al., 2007; Lee and Everitt, 2008a,b,c; Milton et al., 2008a, 2011). Moreover, studying the amnestic effects of propranolol will inform whether the reconsolidation of goal-tracking memories is more similar to conditioned reinforcement or autoshaping/PIT.

#### Conditioned place preference

The most widely-used paradigm in studies of appetitive memory reconsolidation is, in fact, CPP. CPP is a form of Pavlovian conditioning used to measure the rewarding and motivational effects of objects or experiences, and is used in preclinical studies of drug addiction. The CPP apparatus has two distinct chamber contexts, by pairing a certain context within the CPP apparatus with a rewarding drug such as cocaine, amphetamine or morphine, the animal will show preference for the context associated with the drug US and these memories have been demonstrated to undergo reconsolidation (Zhai et al., 2008; Robinson et al., 2011a; Alaghband and Marshall, 2012; Wu et al., 2012a,b). CPP may be supported by context-reward associations, but the contribution of discrete cue-reward associations cannot be discounted (Ito et al., 2008). The disruption of memory reconsolidation in CPP paradigms can be achieved by hippocampal-targeted treatments (Sakurai et al., 2007; Taubenfeld et al., 2010) and intra-BLA protein synthesis inhibition (Milekic et al., 2006) suggesting that context-reward memories do undergo reconsolidation.

The wide use of CPP in reconsolidation studies has resulted in a much greater mechanistic understanding compared to other appetitive memory paradigms (see Sorg, 2012 for review). Here, we will limit our analysis to those mechanisms previously covered in the present review. As noted above, CPP memory reconsolidation has been disrupted by translational inhibitors infused into various neural loci (Milekic et al., 2006; Li et al., 2008, 2010; Yang et al., 2011).

NMDA receptors have been demonstrated to play a functional role in CPP memory reconsolidation. Systemic MK-801 administration was shown to attenuate CPP with cocaine (Brown et al., 2008; Alaghband and Marshall, 2012) and amphetamine (Sadler et al., 2007) rewards. Moreover, for amphetamine CPP, infusion of AP5 directly into the hippocampus impaired memory reconsolidation (Sakurai et al., 2007), although other neural loci have not been investigated.

Propranolol has also been widely employed in studies of CPP memory reconsolidation. Propranolol impaired memory reconsolidation in CPP for cocaine (Phillips and Ledoux, 1992; Bernardi et al., 2006; Otis et al., 2013); morphine (Robinson and Franklin, 2007a; Wu et al., 2012b) and social reward (Achterberg et al., 2012). However, one study of ethanol CPP in mice failed to find evidence for a propranolol-mediated disruption of memory reconsolidation (Font and Cunningham, 2012). Moreover, the critical loci of action of systemically-applied propranolol also remain unclear. In a study of morphine CPP, direct infusions of propranolol into the BLA did not replicate the disruptive effect of local protein synthesis inhibition (Wu et al., 2012b). In contrast, infusion of propranolol into the BLA (as well as the mPFC) did impair memory reconsolidation in a cocaine CPP setting (Otis et al., 2013), a finding that is further supported by the observation that infusions of both α- and β-adrenergic receptor antagonists into the BLA also disrupted cocaine CPP reconsolidation (Bernardi et al., 2009). Given the disruptive effects of these selective adrenergic receptor antagonists, it appears likely that intra-BLA infusions of propranolol would also impair cocaine CPP reconsolidation. Perhaps, then, the dependence of CPP reconsolidation upon BLA adrenergic signaling is related to the nature of the reward used to condition the place preference.

It appears that drug history is an important factor as to whether a CPP memory is affected by reconsolidation disrupting drugs. In drug naïve rats both propranolol and the GABA_A_ agonist midazolam disrupted CPP when administered following reactivation of morphine CPP memory. However, this disruption was not observed in rats with a chronic morphine exposure history, even after 10 days of withdrawal (Robinson et al., 2011a). Similar discrepancies were observed in cocaine-CPP whereby a repeated propranolol administration following reactivation exposures to the drug-paired and unpaired places were required to disrupt preference to the cocaine paired side (Fricks-Gleason and Marshall, 2008).

#### Instrumental behaviors

Instrumental learning entails associating performance of a specific action, such as pressing a lever, to obtain a rewarding outcome such as food. Thus, an association forms between the presence of operant manipulanda (i.e., lever), an action (pressing the lever) and the outcome such as a food reward. While the above paradigms all have clear Pavlovian memory components, instrumental learning is separate in being conceptually distinct from Pavlovian conditioning. Moreover, there remains no definitive demonstration that the memories underlying instrumental behavior undergo reconsolidation. While protein synthesis in the nucleus accumbens is essential for the consolidation of instrumental learning (Hernandez et al., 2002), systemic protein synthesis inhibition failed to impair the reconsolidation of a well-learned instrumental response (Hernandez and Kelley, 2004). Moreover, even when Pavlovian memory reconsolidation was impaired by MK-801, the underlying instrumental behavior remained unaffected (Lee and Everitt, 2008a,b,c; Milton et al., 2008a). However, that is not to say that all memory representations influencing instrumental behavior do not undergo reconsolidation. Post-training changes in outcome values result in updating of the incentive value of rewards, and animals will alter their behaviors accordingly (Dickinson and Balleine, 1990; Balleine and Dickinson, 1992). Lesions to the BLA leave performance insensitive to outcome devaluation (Blundell et al., 2001) and evidence suggests that the BLA is the locus of consolidation and reconsolidation of changes in incentive value (Wang et al., 2005). This was established by infusion of the protein-synthesis inhibitor anisomycin following devaluation of a food reward in an instrumental conditioning paradigm. Anisomycin infused into the BLA abolished changes in the value of the food reward produced by incentive learning, so impaired differential responding controlled by outcome value. In a similar manner, performance of spatial task in a radial arm maze has been demonstrated to be sensitive to NMDA antagonism following memory reactivation (Przybyslawski and Sara, 1997). This task contained an instrumental element whereby rats must acquire a response (direction)—outcome (food reward) association, therefore, in this setting, aspects of instrumental memories may undergo reconsolidation.

#### Summary—appetitive memory

Similar to aversive memories, memory reconsolidation has been demonstrated in a number of appetitive memory settings. These are focussed on Pavlovian appetitive memories, as it remains to be determined whether or not instrumental memories undergo reconsolidation. Appetitive memory reconsolidation appears, like aversive memory reconsolidation, to be fundamentally dependent upon NMDARs, although there is little evidence for local requirement for NMDAR activity in specific neural loci. The picture for β-ARs is more mixed, with amnestic effects of propranolol observed in some, but not all, appetitive memory reconsolidation settings. The underlying cellular mechanisms on appetitive and aversive memories will be further discussed in the following section.

#### Cellular mechanisms of aversive and appetitive memory reconsolidation

At the cellular level, it is important to consider again the canonical dependence of memory reconsolidation upon *de novo* protein synthesis (Nader and Hardt, 2009). The literature on aversive memory reconsolidation reveals some interesting discrepancies in relation to the effects of anisomycin. We have seen examples; albeit involving cross-study comparisons, suggesting that memory reconsolidation can be disrupted by some treatments even when infusion of anisomycin into the same brain region is ineffective (Boccia et al., 2007, 2010; Jobim et al., 2012). Coupled with the criticism that anisomycin and other global protein synthesis inhibitors have effects beyond the inhibition of translation (Rudy et al., 2006), an analysis of the common disruptive effect of protein synthesis inhibitors on memory reconsolidation may not be particularly informative. Moreover, it might be argued that an amnestic effect of protein synthesis inhibition is not necessary to conclude the existence of memory reconsolidation. This is particularly important from an interpretative perspective for appetitive memory reconsolidation, for which protein synthesis inhibitors are seemingly less widely employed than pharmacological antagonists (perhaps due to the potential translation application of addictive drug memory reconsolidation impairments).

We and others have employed antisense oligodeoxynucleotides selectively to inhibit the synthesis of specific proteins. This approach provides further mechanistic information in relation to memory reconsolidation and avoids some of the problems associated with the use of protein synthesis inhibitors. In particular, we have focussed on knocking down the expression of the immediate-early gene Zif268, which had previously been associated with retrieval-related plasticity (Hall et al., 2001; Thomas et al., 2004). Using such an approach, we and others have demonstrated that infusion of Zif268 antisense oligodeoxynucleotides impairs memory reconsolidation. This is the case for discrete cued fear memories and aversive conditioned withdrawal memories in the BLA (Lee et al., 2005; Hellemans et al., 2006; Maddox et al., 2011) and contextual fear memories in the hippocampus (Lee et al., 2004; Barnes et al., 2012). Moreover Zif268 knockdown in the BLA impaired the reconsolidation of the appetitive memories underlying conditioned reinforcement (Lee et al., 2005, 2006a), and in both the BLA and nucleus accumbens core impaired the reconsolidation of cocaine CPP (Theberge et al., 2010). These studies indicate an apparent universality of the requirement for functional Zif268 as a transcription factor in the reconsolidation of both aversive and appetitive memories.

Moreover, there is now an established link between the cell surface signaling at the NMDA receptor and the upregulation of Zif268 in memory reconsolidation. In contextual fear memories, AP5 infusion into the dorsal hippocampus both impaired reconsolidation and attenuated the reactivation-induced upregulation of Zif268 (Lee and Hynds, 2012). This pattern of results replicated that observed in the BLA for cocaine conditioned reinforcement (Milton et al., 2008a,b). Moreover, when the NMDA receptor partial agonist D-cycloserine was infused into the BLA, the reconsolidation of the CS–cocaine memories underlying relapse behavior was potentiated, as was the reactivation-induced upregulation of Zif268 (Lee et al., 2009). Therefore, memory reconsolidation can be bidirectionally modulated by actions at the NMDA receptor, in processes that seemingly rely upon downstream Zif268 expression. When and how other mechanisms, such as β-adrenergic activation, are functionally recruited remains to be clarified. However, it appears that both aversive and appetitive memories are modulated by similar cellular processes.

## When do memories undergo reconsolidation?

As illustrated by the present uncertainty concerning whether or not the memories underlying instrumental behavior undergo reconsolidation, the failure to observe reconsolidation deficits might results from one of three factors. First, the amnestic treatment may be inappropriate or ineffective at disrupting the reconsolidation process. However, given the seemingly-universal requirement for *de novo* protein synthesis, NMDA receptor activation or Zif268 expression, and the failure of inhibition of any of these mechanisms to impair instrumental memory reconsolidation (Hernandez and Kelley, 2004; Lee et al., 2006a; Lee and Everitt, 2008a; Milton and Everitt, 2010), this may be an unlikely explanation. Second, the behavioral parameters used may be ineffective at destabilizing the memory, thereby rendering the amnestic treatment without any behavioral effect. Finally, the memory may genuinely not undergo reconsolidation under any conditions.

The mechanisms of memory destabilization are beginning to be delineated. The first study was conducted in the auditory fear conditioning setting, revealing a double dissociation between memory destabilization and memory expression. Critically, infusion of NMDA receptor antagonists into the BLA protected against reactivation-dependent amnesia (Ben Mamou et al., 2006), reflecting a prevention of memory destabilization. As the memory is not destabilized, it does not require reconsolidation and therefore, the amnesic agent is rendered ineffective.

Several other mechanisms of destabilization have been identified, principally in studies of contextual fear conditioning. These include signaling at L-type voltage-gated calcium channels and cannabinoid CB1 receptors (Suzuki et al., 2008) and synaptic protein degradation (Lee et al., 2008) in the dorsal hippocampus. This leads to the question of whether the mechanisms of destabilization are conserved across neural loci, and it appears that there are discrepancies. As described above, AP5 impairs destabilization of tone fear memories in the amygdala (Ben Mamou et al., 2006), but it impairs reconsolidation both when infused into the hippocampus in a contextual fear setting (Lee and Hynds, 2012) and indeed also when infused into the BLA in a study appetitive conditioned reinforcement (Milton et al., 2008a,b). Similarly, there is controversy over the role of the proteasome inhibitor lactacystin (that prevents protein degradation), which prevented memory destabilization in cocaine-CPP (Ren et al., 2013) as well as contextual fear (Lee et al., 2012) studies. However, infusions of lactacystin into the dorsal hippocampus impaired spatial memory reconsolidation, rather than destabilization, in the water maze (Artinian et al., 2008). Therefore, the differential involvement of synaptic protein degradation in memory destabilization and destabilization occurs both across neural loci and also for different memory types within the same brain region (the dorsal hippocampus).

Recently, we have demonstrated a functional involvement of midbrain dopaminergic signaling in the destabilization of appetitive goal-tracking memories (Reichelt et al., 2013). Phasic midbrain dopamine signals code for prediction error signals that regulate learning. Blocking these putative prediction error signals through dysregulation of VTA function protected a reactivated goal-tracking memory from being disrupted by post-reactivation injections of MK-801. It remains to be determined whether prediction error signals are similarly functionally necessary for the destabilization of aversive memories.

The consideration of whether a memory is successfully destabilized leads us to the concept of boundary conditions. As illustrated by the contrast between our results and those of Blaiss and Janak (2007) in the context of Pavlovian conditioned approach, there are actually numerous observations that within an experimental paradigm, memories do undergo reconsolidation, but not always. Therefore, a description of these boundary conditions is important both for the understanding of the reconsolidation process and for the appropriate application of any reconsolidation-based therapeutic intervention. We have recently argued that the different boundary conditions might be unified under the notion that memory retrieval must result in memory modification in order to trigger memory reconsolidation (Lee, 2009). However, here we will discuss whether the underlying phenomena of the boundary conditions generalize across paradigms and valence of conditioning.

### Reconsolidation vs. extinction

Typically, in studies of Pavlovian memory reconsolidation, memory reactivation and destabilization is achieved by exposure to the CS (whether it is discrete or contextual) in the absence of the US [memories can also be reactivated by exposure to the US. e.g., Schneider and Sherman (1968), but this is rarely used]. Operationally, therefore, memory reactivation actually consists of a brief extinction training session. The behavioral impact of extinction training is to diminish subsequent memory expression (Bouton and King, 1983; Bouton, 2002, 2004; Quirk, 2002), which under most circumstances results from new inhibitory learning (Zimmer-Hart and Rescorla, 1974; Garcia, 2002; Delamater, 2004). It may be surprising; therefore, that amnestic treatment does not impair such new inhibitory learning, thereby resulting in preserved memory expression. In fact, this is observed, but only under conditions that favor extinction over reconsolidation.

The competition between reactivation/reconsolidation and extinction and their opposing effects on subsequent memory expression has been conceptualized within a “trace dominance” framework (Eisenberg et al., 2003). That is, the memory trace that is dominantly activated by the reactivation/extinction session is the one that is impaired by amnestic treatment. This may be due to there being competition between the CS-US memory and the extinction CS-noUS memory for “cellular plasticity resources” when the memory is retrieved. Therefore, if extinction is dominantly engaged, amnestic treatment will impair extinction to preserve subsequent memory expression. In contrast, if reconsolidation is the dominant trace, then memory destabilization will be disrupted, leading to diminished memory expression. It appears that the parametric factors that determine the balance between reactivation and extinction are the strength of training and the extent of unreinforced stimulus exposure at memory retrieval. Eisenberg et al. (2003) demonstrated using CTA in rats that in a memory generated from 1 day of training, extinction was dominantly engaged by memory retrieval and was impaired by anisomycin infusion into the insular cortex. However, when training was more intense, the same retrieval and anisomycin procedure resulted in disruption of reconsolidation. A similar pattern of results was observed in fear conditioning with medaka fish, whereby competition between the original CS-US trace and a new CS-noUS trace arises and the outcome of amnestic treatment depended on the intensity of the original training and the number of extinction trials. Additionally, Pedreira and Maldonado (2003) demonstrated in *Chasmagnathus* crabs that treatment with the protein synthesis inhibitor cycloheximide can either disrupt or preserve the old memory, reflecting impairments of reconsolidation and extinction, respectively, depending on the duration of context re-exposure at memory reactivation. Finally, the concept of trace dominance is not limited to the mnemonic impact of protein synthesis inhibition, as the same pattern of results has been observed in auditory fear conditioning using systemic injections of the NMDA receptor antagonist MK-801 (Lee et al., 2006b).

While the competition between reconsolidation and extinction suggests that brief unreinforced re-exposure to training stimuli is optimal to engage reconsolidation, there is also a minimum level of re-exposure required successfully to destabilize a memory. In a contextual fear conditioning setting, Suzuki et al. (2004) demonstrated that the duration of context re-exposure during memory reactivation was crucial in determining which of three functional outcomes of anisomycin treatment was observed. Following contextual fear conditioning, systemic anisomycin was administered to mice prior to re-exposure to the context for 0 (non-reactivated control), 1, 3, or 30 min. Fear responding was subsequently greatly reduced, reflecting an impairment in memory reconsolidation, in the mice re-exposed to the context for 3 min, but not 0, 1, or 30 min. The contrast with the outcome of the 30-min re-exposure, which revealed an impairment of extinction, replicates the competition between reconsolidation and extinction. Moreover, the lack of any behavioral effect of anisomycin with the 1-min re-exposure indicates that there is also a minimum amount of re-exposure necessary to reactivate and destabilize the memory. Therefore, there appears to be a parameter space between the minimum re-exposure and extinction, in which memories are successfully destabilized.

The competition between reconsolidation and extinction has not, however, been universally observed. In contrast to these previous studies showing that reactivation can evoke either reconsolidation or extinction depending on the duration of re-exposure, Duvarci et al. (2006) sought to establish whether extinction consolidation prevented a reactivated auditory fear memory from undergoing reconsolidation. It was observed that intra-BLA anisomycin disrupted reconsolidation regardless of the duration of stimulus re-exposure and whether or not extinction was functionally engaged, suggesting that extinction is not a sufficient condition to prevent reconsolidation in the amygdala. The reason for the discrepancy between the studies by Lee et al. (2006b) and Duvarci et al. (2006) is not currently clear. While not identical, the behavioral procedures are similar, leaving the amnestic treatment as the likely explanatory factor. Duvarci et al. (2006) suggest that protein synthesis in the amygdala is not necessary for extinction. This doesn't, however, explain why the effects of MK-801 do show trace dominance in the study by Lee et al. (2006b).

Notwithstanding the complication of the study by Duvarci et al. (2006), trace dominance has been relatively widely demonstrated across aversive paradigms. We have recently extended this observation to appetitive Pavlovian memories. In an appetitive goal-tracking setting, varying the extent of training while keeping the parameters of memory reactivation constant revealed contrasting effects of MK-801 (Reichelt and Lee, 2012). A short training period followed by reactivation resulted in extinction being impaired and MK-801 injection prior to reactivation following a longer training period disrupted reconsolidation.

### Memory strength

The strength of a memory can be modulated through the extent of training. We have already seen that the strength of training has an important impact upon the parameters required to reactivate the memory successfully (Eisenberg et al., 2003; Eisenberg and Dudai, 2004). However, we have also seen that the parameter space for memory reactivation is constrained by more than just the engagement of extinction, with there also appearing to be a minimum amount of stimulus re-exposure that is necessary to reactivate a memory (Suzuki et al., 2004). In the standard single footshock conditioning procedure of Suzuki et al. (2004), 3 min of context re-exposure successfully induced memory reconsolidation. However, when conditioning was increased to 3 footshocks, 3 min of context re-exposure was no longer sufficient to reactivate and destabilize to the stronger contextual fear memory. Instead, 10 min context re-exposure was necessary for pre-reactivation anisomycin to impair memory reconsolidation. These findings suggest that while stronger contextual fear memories are more difficult to destabilize, they do still undergo reconsolidation under suitable behavioral conditions. However, it remains possible that very strong memories do not undergo reconsolidation under any circumstances.

There is some evidence in cued fear conditioning that memory strength places an absolute, if transient, boundary on memory reconsolidation, rather than simply constraining the parameter space for memory reactivation (Wang et al., 2009). While a single CS re-exposure successfully destabilized a memory conditioned through one CS–US pairing, neither one nor five CS re-exposures were effective at reactivating a stronger fear memory induced by 10 CS–US pairings. While it is impossible to discount the possibility in any study that a slightly different reactivation method might have been successful, the results indicated that the stronger memory appeared to be resistant to memory destabilization regardless of the parameters of memory reactivation. However, this boundary condition of memory strength was itself constrained by a further boundary condition of memory age. The strong memory was able to be destabilized, and its reconsolidation disrupted, if a period of 30 days was allowed to elapse between conditioning and reactivation. Wang et al. (2009) explained this pattern of results by showing that the strong conditioning down-regulates of the expression of NR2B receptor subunits within the BLA. As described previously, BLA NR2B-containing NMDA receptors are important for the destabilization of CS-fear memories (Ben Mamou et al., 2006), and their downregulation by strong conditioning results in the memory being resistant to destabilization, thereby causing insensitivity to post-reactivation anisomycin infusions. In addition, the dorsal hippocampus was demonstrated to be important in the expression of NR2B receptors in the BLA, whereby pre-training dHPC lesions prevented the downregulation of NR2B, allowing strong memories to undergo reconsolidation within the BLA 2 days post-training (Ben Mamou et al., 2006). However, the observation that older, strong CS–fear memories are more vulnerable to reconsolidation impairments is not widely observed, as discussed later.

Memory strength has not typically been observed as a constraint on memory reconsolidation in appetitive conditioning settings. Reconsolidation deficits have been widely observed across appetitive conditioning paradigms, with little published evidence documenting any difficulties in reactivating memories. Some examples of the requirement for specific parameters of memory reactivation include the apparent necessity for the presentation of the US in selected studies of CPP (Milekic et al., 2006), however, reactivation dependent amnesia was evident without US presentation in other CPP paradigms (Robinson et al., 2011a). Moreover, even seemingly very strong appetitive Pavlovian memories can be reactivated and their reconsolidation disrupted. For example, a CS–cocaine memory conditioned through around 500 pairings was destabilized by 30 CS presentations at reactivation (Lee et al., 2006a). We have recently demonstrated, however, that memory strength does provide a boundary condition on appetitive memory reconsolidation. This was shown in a Pavlovian goal-tracking procedure with food reward, in which reconsolidation was impaired by MK-801 when rats were given 6 days of training and three presentations of each CS at reactivation. However, when training was increased to 12 days, no disruption of reconsolidation was observed with the same three presentations of each CS at reactivation (Reichelt and Lee, 2012). This pattern of results is, therefore, analogous to that observed by Suzuki et al. (2004) in their contextual fear memory setting. However, we do not yet know whether some other magnitude or method of memory retrieval would have successfully destabilized the strong goal-tracking memory (including increasing the interval between training and reactivation), or if the strong 12-day memory is completely resistant to memory destabilization.

### Memory age

As time passes, a new memory becomes increasingly stable by the process of consolidation. However, the stability of this memory can be disrupted by reconsolidation impairments as described previously. Milekic and Alberini (2002), using an inhibitory avoidance task, observed that a recent, young, memory was most likely to be disrupted and were able to demonstrate a temporally-graded decrease in the susceptibility of a reactivated memory to undergo reconsolidation. Thus, memories were more likely to become disrupted at 2 or 7 days post-training, than 14–28 days post-training. This is supported by studies using medaka fish by Eisenberg and Dudai (2004), which found that older memories in medaka became resistant over time to post-reactivation interference, as these older fear memories were less likely to be disrupted by sodium channel blocker amnestic agent application than new memories. In contrast, Debiec et al. (2002) infused anisomycin into the hippocampus to block reconsolidation 3, 15, and 45 days post-training, expecting that at 45 days reactivation of a hippocampally independent memory would not undergo reconsolidation. In contrast to this prediction, reactivation-dependent amnesia was not temporally graded and was observed at each time point. This supported the prior observation by Nader et al. (2000a,b) that a cued fear memory will still undergo reconsolidation 14 days post-training and is susceptible to disruption by intra-BLA infusions of protein-synthesis inhibitors.

Given the difficulty of drawing conclusions from comparisons across studies, perhaps the most compelling evidence for the manner in which memory age impacts upon memory reconsolidation comes again from Suzuki et al. (2004). Using contextual fear conditioning in mice, anisomycin treatment prior to a 3 min CS re-exposure procedure induced reconsolidation of 1- and 3-week-old memories but not 8-week-old memories. However, increasing the duration of the re-exposure period to 10 min made 8-week-old memories susceptible to disruption by anisomycin (Suzuki et al., 2004). These findings support the notion of a temporal gradient in the activation of reconsolidation processes, however, these older memories are still susceptible to reconsolidation with adjustments to the reactivation parameters. Thus, it is easier to destabilize younger than older memories, however, these older memories can be destabilized with greater re-exposure periods (Suzuki et al., 2004). In the inhibitory avoidance study by Milekic and Alberini (2002) the reactivation exposure was not varied, and thus the possibility that older inhibitory avoidance memories do reconsolidate under certain circumstances cannot be discounted. While the tendency of older memories to be more difficult to destabilize is commonly observed as described above, it should be noted that the study by Wang et al. (2009) shows the opposite pattern. With a strongly-conditioned auditory fear memory, memory destabilization was more easily achieved 30 days after conditioning than after 2 days.

Fewer studies have studied the effect of memory age as a boundary condition in an appetitive setting. Wouda et al. (2010) demonstrated that following a 3 week abstinence period following alcohol self-administration training, propranolol (and to some extent MK-801) reduced alcohol seeking over a series of three post-training tests. Similarly, propranolol administration following reactivation reduced sucrose seeking after a 3 week interval between training and reactivation of the memory (Diergaarde et al., 2006) indicating that appetitive sucrose seeking memories could be disrupted following a long post-training interval. Lee et al. (2006a) demonstrated that both older and more recent CS–cocaine memories in rats were disrupted following Zif268 antisense infusion into the BLA, indicating that in a second-order reinforcement maintained cocaine-seeking protocol, memory age did not form a boundary condition on memory reconsolidation. However, given that none of these studies varied the parameters of memory reactivation, it remains possible that the stimulus re-exposure used was sufficiently extensive to overcome the effect of memory age. It might be predicted tentatively, therefore, that more limited stimulus re-exposure would be sufficient only to destabilize younger appetitive memories.

### Summary—when do memories undergo reconsolidation?

Boundary conditions exist for aversive memories in which reconsolidation is dependent on the memory strength, age and the boundary between reconsolidation and extinction evoked during the reactivation of a memory. As described previously, notable failures in memory reconsolidation have been typically in an appetitive setting—in the case of instrumental memories in rats demonstrating insensitivity to protein synthesis inhibitors following reactivation (Hernandez and Kelley, 2004). However, our recent work has indicated that appetitive Pavlovian goal-tracking memories, previously assumed to not undergo reconsolidation, are in fact sensitive to NMDA antagonist application following reactivation and clear boundary conditions to allow reconsolidation have been identified (Reichelt and Lee, 2012). Thus, failures to observe and impair reconsolidation may simply be due to reactivation conditions being insufficient to destabilize the memory. Recently, optogenetic techniques have been used to induce phasic dopaminergic signals in the VTA, thereby providing direct functional evidence for the involvement of these signals in learning utilizing extinction and blocking paradigms (Steinberg et al., 2013). Such an experimental approach would be predicted also to enable appetitive memory destabilization under behavioral (boundary) conditions that do not normally engage reconsolidation. Given that experimental impairments in appetitive memory reconsolidation represent a potential treatment for drug addiction (Milton and Everitt, 2010), it is particularly important that boundary conditions on appetitive memory reconsolidation continue to be studied. The little evidence to date does, however, suggest that the same principles of memory strength, age and extinction apply equally to appetitive memories as they do to aversive memories.

## Translational applications—from animals to humans

The animal studies detailed in this review are applicable to the use of reconsolidation-disrupting drugs in the treatment of the maladaptive memories that underpin drug addiction and PTSD. Drug memories are appetitive in nature whereas traumatic memories are highly aversive. Early research with rats indicated the importance of β-AR signaling following reactivation of emotional memories and was highlighted as a potential therapeutic strategy for PTSD (Przybyslawski et al., 1999). In humans, disrupting reconsolidation of reactivated traumatic memories may offer a novel pharmacological treatment for PTSD. Although systemic treatment with NMDAR antagonists and protein synthesis inhibitors have proven useful in disrupting memory reconsolidation in animal models, the side effects associated with these drugs makes them less suitable for use in humans. In contrast, propranolol is approved for the treatment of anxiety and hypertension in humans.

Preclinical studies such by Kindt et al. (2009), see also (Soeter and Kindt, 2012; Kindt and Soeter, 2013) have demonstrated the amnestic effect of propranolol on human fear memory reconsolidation. In the study by Kindt et al. (2009), humans were conditioned to associate fear-relevant stimuli (images of spiders) with electric shocks. Oral administration of propranolol prior to memory reactivation in humans erased the behavioral expression of the fear memory 24 h later and prevented the return of fear, whereas fear was still expressed in the control (placebo drug) group. Subsequently, it has been shown that these behavioral effects correlate with a decrease in amygdala activation (Schwabe et al., 2012). These studies indicate that manipulation of β-adrenergic signaling could provide a potential therapeutic mechanism in the treatment of human anxiety disorders, and recently clinical trials using propranolol for PTSD treatment have being conducted with promising results (Brunet et al., 2008, 2011b; Poundja et al., 2012).

Propranolol treatment is particularly suited to PTSD due to its post-reactivation systemic efficacy in pre-clinical studies (Debiec and Ledoux, 2006). However, in human clinical trials using propranolol to treat PTSD, pre-reactivation administration is used (Brunet et al., 2008, 2011b; Poundja et al., 2012). It has been argued by some that reconsolidation-disrupting treatments should only be administered following memory reactivation, so as not to influence the process of memory retrieval (Schiller and Phelps, 2011). However, it is discussed by Brunet et al. (2011a), with respect to the limited “reconsolidation window” and that the bioavailability of oral propranolol peaks about 90 min following administration, protocols using post-reactivation propranolol are therefore, vulnerable to negative results due to the limited effects on protein synthesis by time the drug reaches its full effect in the human brain, so pre-reactivation administration may be best suited.

In appetitive settings, data from rodent studies indicate that memories for both drug and non-drug CS-US associations are dependent on NMDAR, protein synthesis and beta-adrenergic receptor-mediated signaling for their reconsolidation (Lee et al., 2005, 2006a, 2009; Diergaarde et al., 2006; Lee and Everitt, 2008a; Milton et al., 2008a,b, 2012; Milton and Everitt, 2010; Reichelt and Lee, 2012). As described previously, propranolol has been approved for therapeutic use in humans and therefore, poses as a potential treatment for drug addiction and some forms of obesity where food has maladaptive addictive properties. This might be viewed as being relevant as propranolol was able to disrupt sucrose seeking behaviors following a long post-training interval in a well-established memory (Diergaarde et al., 2006). However, as reviewed earlier, propranolol is less effective at disrupting the different appetitive memory representations that contribute to relapse behavior than is the NMDA receptor antagonist MK-801, and there was little evidence for a beneficial long-lasting impact in an exploratory clinical study of propranolol-induced reductions of cocaine craving (Saladin et al., 2013). Therefore, further studies are required at the preclinical level in order to identify amnestic treatments that have both the clinical safety of established drugs such as propranolol, but with the reconsolidation-impairing efficacy of MK-801.

An alternative approach to the pharmacological disruption of memory reconsolidation is to manipulate the aversive CS-US memory behaviorally during the reconsolidation window. This makes use of a non-invasive “retrieval-extinction” design, whereby a reactivated memory is seemingly updated with an extinction (or CS-noUS) memory. Initially this effect was observed in cued fear conditioning studies in both rats (Monfils et al., 2009) and humans (Schiller et al., 2010). In both cases, the reduction in fear memory expression was long-lasting and did not recover with reminders. While there have been notable failures to replicate the retrieval-extinction effect in the cued fear conditioning setting (Chan et al., 2010; Auber et al., 2013), the observation has been extended to contextual fear memories (Flavell et al., 2011; Rao-Ruiz et al., 2011). Moreover, retrieval and extinction diminishes appetitive conditioned reinforcement for sucrose reward (Flavell et al., 2011) and CPP for morphine and cocaine rewards (Xue et al., 2012). The latter study of drug addiction even showed efficacy in reducing cue-induced heroin craving in heroin addicts. However, the conceptual basis of the effect of the retrieval-extinction paradigm has been questioned with respect to whether the procedure simply deepens the learning that normally happens during extinction, rendering responding less vulnerable to reinstatement. Recently, (Millan et al., 2013) demonstrated that retrieval-extinction may not always be protective of reinstatement when testing conditions involves contingent presentations of the reinforcer. In this study the retrieval—extinction manipulation significantly increased the motivation of animals to respond for and consume the drug relative to standard extinction training when tested under a progressive ratio schedule. In addition, the order in which memory retrieval and extinction occurs should be crucial, as retrieval trial must occur before extinction training in order to reactivate the original memory and allow the new extinction learning to be incorporated prior to reconsolidation (Tronson and Taylor, 2007; Nader and Hardt, 2009). However, this study demonstrated that when a short retrieval session (10 min) was performed following the extended extinction session (50 min), yielded a resistance to reinstatement which is conceptually not possible within the current framework of reconsolidation, whereby the memory requires destabilization prior to the incorporation of new information (Millan et al., 2013).

Therefore, there is preliminary evidence supporting a clinically-beneficial effect of reconsolidation-based treatment strategies for both PTSD (propranolol-induced reconsolidation impairments) and drug addiction (purely behavioral retrieval-extinction effects). The common dependence of the two interventions upon memory destabilization and reconsolidation demands a comparative analysis of their relative efficacy in the search for the balance between treatment efficacy and potential drug side-effects.

### Conflict of interest statement

The authors declare that the research was conducted in the absence of any commercial or financial relationships that could be construed as a potential conflict of interest.
